# The Population Structure of *Pseudomonas aeruginosa* Is Characterized by Genetic Isolation of *exoU+* and *exoS+* Lineages

**DOI:** 10.1093/gbe/evz119

**Published:** 2019-06-07

**Authors:** Egon A Ozer, Ekpeno Nnah, Xavier Didelot, Rachel J Whitaker, Alan R Hauser

**Affiliations:** 1 Division of Infectious Diseases, Department of Medicine, Northwestern University Feinberg School of Medicine; 2 Lurie Children’s Hospital, Chicago, Illinois; 3 School of Life Sciences and Department of Statistics, University of Warwick, Coventry, United Kingdom; 4 Department of Microbiology and the Carl R. Woese Institute of Genomic Biology, University of Illinois, Urbana-Champaign; 5 Department of Microbiology-Immunology, Northwestern University Feinberg School of Medicine

**Keywords:** population structure, recombination, whole-genome phylogenetics, microbial evolution, accessory genome, *exoU*, *exoS*

## Abstract

The diversification of microbial populations may be driven by many factors including adaptation to distinct ecological niches and barriers to recombination. We examined the population structure of the bacterial pathogen *Pseudomonas aeruginosa* by analyzing whole-genome sequences of 739 isolates from diverse sources. We confirmed that the population structure of *P. aeruginosa* consists of two major groups (referred to as Groups A and B) and at least two minor groups (Groups C1 and C2). Evidence for frequent intragroup but limited intergroup recombination in the core genome was observed, consistent with sexual isolation of the groups. Likewise, accessory genome analysis demonstrated more gene flow within Groups A and B than between these groups, and a few accessory genomic elements were nearly specific to one or the other group. In particular, the *exoS* gene was highly overrepresented in Group A compared with Group B isolates (99.4% vs. 1.1%) and the *exoU* gene was highly overrepresented in Group B compared with Group A isolates (95.2% vs. 1.8%). The *exoS* and *exoU* genes encode effector proteins secreted by the *P. aeruginosa* type III secretion system. Together these results suggest that the major *P. aeruginosa* groups defined in part by the *exoS* and *exoU* genes are divergent from each other, and that these groups are genetically isolated and may be ecologically distinct. Although both groups were globally distributed and caused human infections, certain groups predominated in some clinical contexts.

## Introduction

*Pseudomonas aeruginosa* is a gram-negative bacterium that is remarkable for its worldwide ubiquity and extensive environmental distribution in soil, water, and plant matter as well as its ability to cause a variety of opportunistic infections in humans. It is a major cause of morbidity and mortality in hospitalized patients and those with cystic fibrosis (CF). In addition to the production of a formidable number of virulence factors, both intrinsic and acquired antibiotic resistance mechanisms contribute to the species’ importance as a human pathogen.

Several previous investigations into the population structure of *P. aeruginosa* have been undertaken. Earlier studies relied on a variety of typing methods, such as gel electrophoresis banding patterns, multilocus sequence typing, or microarray analysis to characterize relationships between groups of isolates (Kiewitz and Tummler 2000; Wiehlmann et al. 2007; Pirnay et al. 2009). As next-generation sequencing has become more affordable and widely available, *P. aeruginosa* population studies have started using whole-genome comparisons between increasing numbers of isolates (Stewart et al. 2011; Freschi et al. 2015; Hilker et al. 2015; Marvig et al. 2015; Williams et al. 2015; Freschi et al. 2019). In these phylogenetic analyses, isolates within the populations examined have generally clustered into two large clades and one small clade. The geographical sources of isolates do not appear to account for these phylogenetic clusters (Wiehlmann et al. 2007; Kos et al. 2015; England et al. 2018). Recent studies showed certain genotypes were found more abundantly in environmental isolates than in human-derived isolates, and vice versa (Wiehlmann et al. 2015; Rutherford et al. 2018). However, the genetic differences underlying the observed population structure and possible mechanisms for these differences have not yet been defined.

Early studies classified *P. aeruginosa* isolates as either cytotoxic or invasive (Fleiszig et al. 1996). It was later discovered that cytotoxic isolates usually secreted the effector protein ExoU by a type III secretion pathway (Finck-Barbançon et al. 1997). ExoU is a patatin-like phospholipase A_2_ enzyme that cleaves lipids within eukaryotic host cell membranes (Phillips et al. 2003; Sato and Frank 2004). In contrast, invasive isolates usually secreted ExoS, which is a bifunctional enzyme with Rho GTPase-activating protein and ADP-ribosyltransferase activities (Barbieri and Sun 2004) that causes multiple effects on eukaryotic cells, including cell rounding and apoptosis (Kaufman et al. 2000; Barbieri et al. 2001). For unclear reasons, the large majority of *P. aeruginosa* isolates contain either the *exoU* or the *exoS* gene, but isolates rarely carry both genes or neither gene (Feltman et al. 2001; Lomholt et al. 2001; Garey et al. 2008; Pirnay et al. 2009; Bradbury et al. 2010). This distinction is of clinical importance, as *exoU+* isolates are associated with more severe infections and higher mortality in acutely infected patients (Finck-Barbançon et al. 1997; Hauser et al. 1998; Schulert et al. 2003; Shaver and Hauser 2004; El-Solh et al. 2012; Pena et al. 2015).

We sought to examine the population structure of a collection of 739 geographically diverse clinical and environmental *P. aeruginosa* isolates using whole-genome phylogenetic analysis. We confirmed that most *P. aeruginosa* isolates fell into one of two large groups based upon the core genome, with rare isolates belonging to one of at least two smaller groups. We showed that core and accessory gene flow between isolates of the same group was much greater than between isolates of different groups, suggesting that the two groups are genetically isolated. We identified core and accessory sequences that were highly discriminatory between the two major groups. In particular, *exoS* was present in nearly all the isolates of one large group and *exoU* in nearly all the isolates of the other large group.

## Materials and Methods

### *Pseudomonas aeruginosa* Isolates

A total of 730 genomic sequences representing all complete *P. aeruginosa* genomic sequences as well as all draft genomic sequence contigs was downloaded from the NCBI FTP site (ftp.ncbi.nlm.nih.gov) on February 3, 2015. Isolate demographic information including continent and country of origin, clinical or environmental source, and CF status of the source patient for clinical isolates was determined, when available, from NCBI BioSample or BioProject entries. In cases where the relevant information was not listed in these resources, associated publications, as listed in the NCBI BioProject entries for the isolates, were manually reviewed for the relevant metadata.

### Previously Unsequenced Environmental Isolates

Nine previously described environmental isolates of *P. aeruginosa* (Feltman et al. 2001) were selected for sequencing. These isolates were streaked from −80 °C frozen stocks, inoculated in Luria-Bertani broth, and grown with shaking overnight at 37 °C. Genomic DNA was extracted from the cultures using the Promega Maxwell 16 instrument (Madison, WI) according to the manufacturer’s instructions. Genomic DNA was sequenced on the HiSeq 2000 platform yielding 101-bp paired-end reads. To maximize assembly quality (Wall et al. 2014), each paired read set was randomly downsampled to obtain estimated 80-fold genome coverage and de novo assembled using Ray v1.7.0 (Boisvert et al. 2010). Assembled contigs smaller than 200 bp were removed from the analysis. Contig sequences were deposited in GenBank under assembly accession numbers GCA_002239415.1, GCA_002239425.1, GCA_002239445.1, GCA_002239465.1, GCA_002239485.1, GCA_002239505.1, GCA_002239535.1, GCA_002239545.1, and GCA_002239565.1.

### Type III Effector, O-Antigen Biosynthesis Locus, and Genomic Island Typing

Reference nucleotide sequences of the type III effector genes *exoU* (locus ID PA14_51530 in strain UCBPP-PA14) and *exoS* (locus ID PA3841 in strain PAO1) were obtained from the Pseudomonas Genome Database (Winsor et al. 2011). Presence or absence of the *exoU* and *exoS* genes was determined by BlastN alignment of the *exoU* and *exoS* nucleotide sequences against the genomic sequences of each isolate using default parameters (Altschul et al. 1990). The contents of *exoS* gene locus were identified using *in silico* polymerase chain reaction (PCR) using an in-house Perl script (https://github.com/egonozer/in_silico_pcr; Last accessed June 2019) to extract sequences between conserved flanking genes PA3840 (ATGCCCCGCCCGACCAGCCC) and *spcS* (TCAGCGTAGCTCTTCGGCGG).

O-antigen biosynthetic gene cluster typing was performed using *in silico* PCR. Given the variability in sizes and the heterogeneity of the contents of the O-antigen biosynthetic locus among strains, we chose the *in silico* PCR approach to identify and isolate the loci contents based on conserved flanking region sequences. Sequences of genes *rpsA* (locus ID PA3162 in strain PAO1) and *tyrB* (locus ID PA3139 in strain PAO1), which are conserved and flank the O-antigen region, were obtained from the Pseudomonas Genome Database (Winsor et al. 2011). The reverse-complement of the first 20 nucleotides of the *rpsA* gene (AGATGGAGAATCAGGGCTAA) were used as the forward primer sequence and the first 20 bases of the *tyrB* gene (CCATCGTCCAGGTCCTGTAG) were used as the reverse primer. *In silico* PCR was performed on each of the genomic sequences allowing for up to one base mismatch and one base insertion or deletion in each primer sequence. When primer sequences were found on separate contigs, sequences from each primer to the respective contig ends were manually joined into a single sequence. The resulting “amplicon” nucleotide sequences were aligned using BLAST against the 21 O-antigen locus nucleotide sequences (Raymond et al. 2002) to assign each locus to one of the 11 possible O-antigen biosynthetic locus groups. When the length of any reference O-antigen locus aligned to the “amplicon” sequence was <90%, BLAST was used to align the whole-genome sequence assemblies against the representative O-antigen locus nucleotide sequences to identify the locus group type. This might occur, for example, in cases where the O-antigen locus spanned multiple contigs such that only the locus ends could be identified by *in silico* PCR.

Markers for specific genomic islands (GIs) were identified by *in silico* PCR using primers described by Morales-Espinosa et al. (2012). Up to one base mismatch and one base insertion or deletion per primer was allowed. An *in silico* PCR result was considered positive if both primer sequences were found on opposite strands on the same contig or if both primer sequences were found on separate contigs, but the distance from the primer sequences to the ends of the contigs each did not exceed the expected amplicon size.

### Variant Detection and Phylogenetic Analyses

The kSNP v2.1.2 program (Gardner and Hall 2013) was used to identify single nucleotide polymorphisms (SNPs) in the core genome. Briefly, kSNP identifies variants among genomes by separating assemblies into k-mers, and identifying k-mers sharing most sequence between isolates but differ by a single nucleotide. For the purposes of this study, the core genome variants were defined as loci found in at least 95% (i.e., ≥702) of the isolates with a variant in at least one of the isolates at that locus. This definition was chosen to minimize the impact on the core genome of a small number of isolates that might have undergone core gene deletion or for which sequencing or assembly errors may have resulted in omission of genetic sequence. All k-mers 21 bp in length were examined, as selected by the Kchooser script included with kSNP.

We chose kSNP for identifying core genome variants and performing phylogenetic analyses for several reasons. First, our data set consisted of assembled genome sequences deposited at NCBI, so we could not use methods to identify variants based on alignments of sequencing reads to a reference. Second, the number of genomes analyzed exceeds the computational limits of other software programs used to align and call variants in assembled genomes. Third, given the variability in assembly qualities and completeness of the genomes used, we thought it important for our analyses to allow some flexibility in the core genome definition to include variants in regions that were present in the large majority of the included isolate assemblies but not necessarily found in every genome. Most available core genome alignment programs will only generate alignments of regions present in 100% of the included isolates. As kSNP can identify single nucleotide variants (SNVs) in assembled genomes, is computationally scalable to analyze large data sets, and allows flexibility in core genome definition, we chose to use this software for variant detection and phylogenetic analyses.

For secondary validation of the tree structures generated using kSNP, a whole-genome alignment method against a reference sequence was used. Each isolate’s genome sequence was aligned to the sequence of *P. aeruginosa* PA14 (accession no. CP000438.1) using nucmer, and SNPs were called from the alignment using show-snps. Both programs are part of the MUMmer software suite version 3.23 (Kurtz et al. 2004). A custom Perl script, nucmer_snp_to_matrix.pl, was then used to filter and arrange SNP loci into a sequence matrix. Any variants against the reference sequence that were within ten bases of each other or within five bases of a contig end were omitted. FastTreeMP v2.1.7 (Price et al. 2010) was used to generate a maximum likelihood phylogenetic tree. Phylogenetic trees in this study were visualized using either FigTree v1.4.3 (http://tree.bio.ed.ac.uk/software/figtree/; Last accessed June 2019) or Evolview (Zhang et al. 2012; He et al. 2016) for different representations and annotations.

### Accessory Genome Characterization

The core genome of the 739 *P. aeruginosa* isolate collection was determined using Spine v0.1.2 (Ozer et al. 2014). Sequence was considered part of the core genome if it was present in at least 703, or 95%, of the isolates with at least 85% sequence identity. The accessory genome of each input isolate was determined using AGEnt v0.1.3 (Ozer et al. 2014). ClustAGE was used to align and group the accessory genomic sequences of all isolates and identify the distribution of accessory genomic elements (AGEs) among the isolates (Ozer 2018). Briefly, AGEs were grouped together by combining accessory sequences from all genomes. Then, starting with the largest AGE, now identified as a representative “bin,” AGE sequences from all other isolates were aligned to the bin using BlastN (Altschul et al. 1990). All AGEs aligning to the bin with at least 85% sequence identity and an *E*-value of at most 1 × 10^−6^ were considered “binned” with the representative AGE bin and removed from the pool of potential bins. If only a fraction of an AGE aligned to a particular bin sequence, the unaligned portion of the AGE was returned to the pool of potential bin sequences. The next longest remaining AGE or partial AGE sequence in the bin pool was then used as a blast query sequence against the database of all AGE sequences. This process was continued until all AGEs had either been binned with a representative AGE or served as a bin representative themselves or were <200 bp in length. As AGEs are often mosaic in composition between isolates, alignments against each bin representative were then parsed to further subdivide bins into “subelements” at positions along the bin sequence where either the number or identities of genomes from which aligning AGEs were found changed. In this way, a bin could be divided into subelements ranging in size from 1 bp up to the length of the representative bin AGE, with each subelement sequence identified as a continuous sequence element present in the accessory genome of at least one input isolate. We chose to use the Spine/AGEnt/ClustAGE approach to characterize the accessory genome of this population as it is well suited for identifying commonalities and differences in genomes from large populations in coding and noncoding sequences alike without a priori knowledge of accessory element sequences.

To assess relative amounts of shared accessory genome sequence between pairs of isolates, we adapted an approach described by Shapiro et al. (2012). Briefly, the Bray–Curtis distance (*d*) of the accessory genome of each pair of isolates was calculated using sizes of shared AGEs at least 100 bp in length. These distances were used as input to Phylip v3.695 (http://evolution.gs.washington.edu/phylip; Last accessed June 2019) to produce a neighbor joining tree. The neighbor joining tree was visualized with FigTree v1.4.2 (Rambaut), and the heatmap from the inverse of the Bray–Curtis distances (1 – *d*) was visualized with R v3.4.1 (R Core Team 2016) using the ComplexHeatmap package v1.15.1 (Gu et al. 2016). Multiple correspondence analysis of AGE distribution was performed using the MCA function of the R package FactoMineR v1.41 (Lê et al. 2008) and visualized using the factoextra package v1.0.5. Pangenome sizes and new genome sizes for random permutations of genomes were calculated as previously described (Ozer et al. 2014).

### Recombination Analysis

To examine patterns of core genome recombination within the population, a 95% core genome multiple sequence alignment was constructed based on the kSNP analysis results. Briefly, we sought to convert the kSNP program output, which is a matrix of variant positions and bases in each isolate, into a multiple sequence alignment representing the distribution of SNVs within a reference genome. We selected PA14 to serve as the reference sequence representing each of the 739 isolates. Then, for each genome, we used information from the kSNP matrix to change bases at core genome positions in PA14 to match the base found at that position in the non-PA14 isolate sequence. The result was an alignment of 739 sequences, each sequence the length of the PA14 whole genome and each representing the sequence of one of the 739 studied genomes at core genome sites. ClonalFrameML v1.11 (Didelot and Wilson 2015) was used to reconstruct recombination events in the full core genome multiple sequence alignment of all 739 isolates, as well as separately among isolates in each of the major groups. The likely origin of each recombination event detected by ClonalFrameML was inferred using similar methods as previously described (Didelot et al. 2009, 2011; Sheppard et al. 2013; Cao et al. 2015) and briefly summarized below. The sequence imported in each recombination event was compared with the imputed sequences of all nodes and leaves in phylogenetic trees of both the recipient group and other nonrecipient groups to determine the minimum genetic distance. For recombination events on terminal branches, comparisons to the leaf under that branch were excluded, whereas for recombination events on nonterminal nodes, all comparisons to nodes and leaves below the recombination event were also excluded. If a recombination event was found to have a minimum genetic distance to sequences in the recipient group below the threshold value, but minimum distance to all nonrecipient groups’ sequences above the threshold value, the importation event was inferred to have originated within the recipient group. Conversely, if the minimum distance to the recipient group was above the threshold, but the minimum distance to one of the nonrecipient groups was below the threshold, the recombination event was inferred to have originated from the nonrecipient group. If no group’s minimum distance was below the threshold, the recombination event’s source was inferred to be external to the population, and if more than one group’s minimum distance was below the threshold, the recombination event’s origin was classified as ambiguous. Based on the estimated mean divergence of imported DNA sequences for the population, that is, the parameter “nu” derived by ClonalFrameML, a threshold distance of 0.002 was chosen. Recombination flow diagrams were produced using GraphViz (http://www.graphviz.org; Last accessed June 2019).

To count polymorphic and fixed variants within and between groups of isolates and perform the McDonald–Kreitman test for each gene, a custom Perl script, MKT_per_gene.pl, was developed. Individual gene alignments were extracted from the whole-genome alignment described above. Polymorphisms found in <5% of all genomes were ignored. A variant was considered fixed if present in at least 98% of genomes in a group.

### Admixture Analysis

The core genome multiple alignment described above was also used for admixture analysis. Hierarchical clustering was performed using the hierBAPS module included with BAPS v6.0 (Cheng et al. 2013) with a maximum cluster number (*K*) of 35. The results of the first level of clustering were then used as input for admixture analysis in BAPS v6.0 using default parameters (Corander and Marttinen 2006; Corander et al. 2008). A gene flow diagram was produced using GraphViz (http://www.graphviz.org).

### Average Nucleotide Identity

Average nucleotide identity (ANI) was calculated for each pair of genome sequences as previously described (Goris et al. 2007). For each combination of genome sequences, both reciprocal ANI values were determined.

### Statistical Analyses

Exact test of goodness-of-fit analyses with Holm corrections for multiple observations was performed in R v3.4.1 (R Core Team 2016).

## Results

### Most *P. aeruginosa* Isolates Segregate into Two Large Phylogenetic Groups

Genomic sequences of 730 *P. aeruginosa* isolates representing all complete and draft genome sequences available as of February 3, 2015, were downloaded from the NCBI FTP server. When available, relevant metadata for each sequenced isolate was collected (supplementary table 1, Supplementary Material online). The number of genomic sequences from isolates identified as clinical in origin (*n* = 615) far exceeded the number identified as environmental in origin (*n* = 57). To increase the representation of environmental isolates in the data set, we sequenced nine additional isolates of *P. aeruginosa* previously collected from environmental sources (Feltman et al. 2001) (supplementary table 1, Supplementary Material online). The total set of assemblies ranged in size from 5,502 to 7,586 kb (median 6,644 kb) and consisted of 1–2,797 contigs per assembly (median 98 contigs). GC content ranged from 65.19% to 66.87% (median 66.20%).

**Fig. 1.**
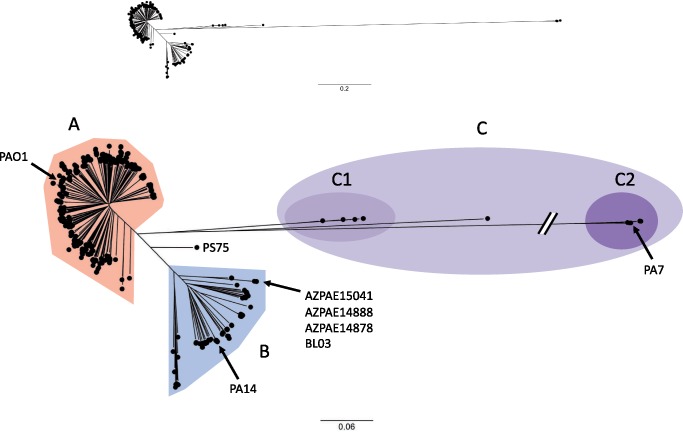
—Population structure of *Pseudomonas aeruginosa* isolates. The upper panel shows a maximum likelihood phylogenetic tree generated from core genome SNP loci in 739 *P. aeruginosa* isolates. The lower panel shows an expanded version of the same phylogenetic tree with a truncated outlier branch. Major branches are indicated by labels and highlighting: Group A (red), Group B (blue), and Group C (purple). Group C isolates are further subdivided into Group C1 (light purple) and Group C2 (dark purple). Several isolates mentioned in the text are indicated. The scale bars represent genetic distances.

Next, core genome SNVs were identified. Core genome SNVs, defined as loci with sequence found in at least 95% (≥703) of the 739 isolates and with a variable base in at least one genome, were identified using kSNP v2.1.2 (Gardner and Hall 2013). kSNP uses a reference-free alignment approach to identify SNV differences between genomic sequences by dividing the genomes into equal length k-mers (all possible stretches of k-consecutive nucleotides) and aligning k-mers from different genome sequences to identify interisolate base differences. This approach has the advantage of not requiring multiple sequence alignments to a single reference genome, which allows for rapid comparisons of large numbers of genomes. The core genome phylogenetic tree was based on 368,212 core SNV loci identified by kSNP (fig. 1) . As has been observed by others (Freschi et al. 2015, 2019; Kos et al. 2015), the large majority of isolates (98%) fell into one of two major groups designated here as “Group A” (541 isolates) or “Group B” (186 isolates). Most of the remaining isolates cluster onto a third branch of the tree, “Group C” (11 isolates), with some of these isolates demonstrating considerable core genome phylogenetic distance from the Group A and Group B isolates. Isolates in Group C were further subdivided into two smaller subclades, Group C1 (five isolates) and the more distant Group C2 (five isolates), with one isolate, CF_PA39, falling between the two groups. The commonly used lab strains PAO1 and PA14 are found in Group A and Group B, respectively (fig. 1). PA7, which has previously been described as phylogenetically distinct from most other *P. aeruginosa* isolates (Roy et al. 2010), is found in Group C2.

To further support the structure of the phylogenetic tree generated by the reference-free kSNP analysis, we used a secondary reference-alignment-based approach. Assemblies were individually aligned to the PA14 genomic sequence using nucmer (Kurtz et al. 2004), and all loci with a variant against PA14 in which a nucleotide position was present in at least 95% of the isolates were combined in a sequence matrix containing 502,674 core genome variant loci. The clade structure of the tree produced from this core genome SNV alignment matrix was similar to the tree generated by kSNP (supplementary fig. 1, Supplementary Material online), supporting the accuracy of the kSNP tree.

Next, the impact of recombination on the core genome phylogenetic tree was examined using ClonalFrameML to identify potential recombination events and reconstruct the phylogeny with corrected branch lengths. The resulting corrected core genome phylogenetic tree showed decreased branch lengths, but the separation of the population into distinct groups remained unchanged (supplementary fig. 2, Supplementary Material online; note scale bar), indicating that the phylogenetic separation of isolates into Groups A and B was not an artifact of recombination.

### Core Genome Recombination Flow Indicates a Barrier to Genetic Exchange between Group A and Group B Isolates

The differentiation of Group A isolates from Group B isolates could result from the two groups evolving in distinct ecological niches or because of physical and/or genetic barriers that limit recombination between these groups (Cohan 2002a; Cadillo-Quiroz et al. 2012; Shapiro et al. 2012). We therefore investigated patterns of core genome recombination among the 739 isolates. First, we used the results of the ClonalFrameML analysis to quantify rates of recombination. In the entire population, the relative rate of recombination was estimated to be about 4-fold less than the mutation rate (*R*/theta = 0.27). However, because each recombination event can convey multiple nucleotide changes, recombination was estimated to contribute more than 2.5-fold more diversity to the population than mutation (*r*/*m* = 2.53, which is the product of *R*/theta, the mean recombination length delta and the mean divergence of imports nu) (table 1). Examination of recombination in Group A strains only showed a higher relative rate of recombination versus mutation than the population as a whole (*R*/theta = 0.55) with an overall greater effect of recombination on diversity (*r*/*m* = 3.69). By contrast, the relative recombination rate was found to be lower within the Group B isolates (*R*/theta = 0.17), but due to a 10-fold higher average length of recombinant regions (delta), the relative contribution of recombination to isolate diversification was much higher in the Group B isolates (*r*/*m* = 8.43). Repeated analyses of random subsets of isolates from each group confirmed the differences in recombination parameters between the groups (supplementary fig. 3, Supplementary Material online). These findings indicate that core genome recombination events are quite common in *P. aeruginosa* but that the nature of these recombination events differ between Group A and Group B isolates.

**Table 1:** Recombination Parameters

Group	# Strains	# Events	*R*/Theta^a^	Delta^b^	Nu^c^	*r*/*m*^d^
All	739	19,965	0.2720	4209.9	0.002206	2.5261
Group A	541	17,993	0.5491	3193.8	0.002103	3.6885
Group B	186	1,635	0.1686	31277.3	0.001598	8.4280
Group C1	5	124	0.1349	1738.3	0.004562	1.0694
Group C2	5	185	0.6390	1480.0	0.001988	1.8800

^a^Relative rate of recombination to mutation.

^b^Mean DNA import length.

^c^Mean divergence of imported DNA.

^d^Relative contribution of recombination versus mutation to diversity.

**Fig. 2.**
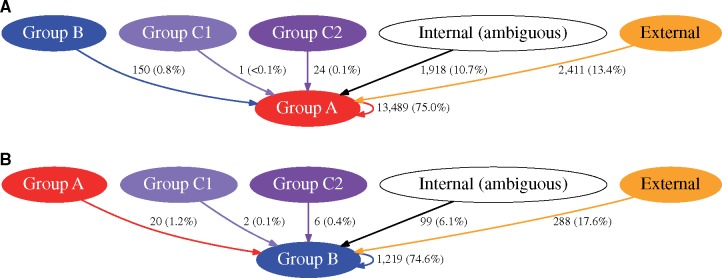
—Core genome recombination between the major groups of *Pseudomonas aeruginosa*. The inferred sources of recombinant regions identified within isolates in Group A (panel *A*) and Group B (panel *B*) are shown. The vectors indicate the direction of recombinant region flow. “Internal (ambiguous)” represents a source of recombinant sequence within Groups A, B, C1, or C2 but for which the source could not be attributed to any one of the groups. “External” represents a source of recombination from outside of Groups A, B, C1, or C2. Vectors are labeled with the number of recombination events originating from each source, and the numbers in parentheses are the percentages of the total recombination events in the destination group represented by each vector.

To further examine whether bacteria in Group A and Group B were evolutionarily independent lineages (i.e., that intergroup recombination events are relatively rare compared with intragroup recombination events), we examined the estimated sources of recombinant sequence. To infer likely recombinant region origins, genetic distances were calculated between recombination event sequences and their corresponding prerecombination sequences as reconstructed by ClonalFrameML in each group of isolates. In Group A isolates, 13,489 (75.0%) of 17,993 recombination events likely originated from within Group A but only 150 events (0.8%) originated from Group B (fig. 2*A*). Similarly, in Group B isolates, 1,219 (74.6%) of 1,635 recombination events likely originated from within Group B but only 20 events (1.2%) originated from Group A (fig. 2*B*). Fewer than 0.5% of recombination events in either Group A or Group B isolates were attributed to a source among Group C1 or Group C2. A total of 10.7% and 6.1% of recombination events in Group A and Group B isolates, respectively, were sequences that likely originated from Groups A, B, C1, or C2, but the source could not be unambiguously assigned to a single group. The remaining recombination events in each group were predicted to be from an “external” origin (i.e., from a source genome outside of Groups A, B, C1, or C2). A limitation of this analysis is that the relatively small number of isolates in Group C1 and Group C2 included in this population may have precluded an accurate estimation of the true overall diversity of this group, potentially causing some recombinant sequences from these groups to be attributed to an external donor source. Recent reports have identified additional isolates that belong to the C subgroups (Freschi et al. 2019), which should allow future studies to better analyze their genomic features. The overall finding of a strong bias toward intragroup recombination relative to intergroup recombination suggests a barrier to cross-group exchange of genetic material (Didelot et al. 2010; Ansari and Didelot 2014), which is consistent with the notion that Groups A and B inhabit distinct ecological niches or that a genetic barrier to recombination exists between them.

To examine the relative distribution of recombinant sequence between major groups in the population, we used BAPS (Corander and Marttinen 2006; Corander et al. 2008) to perform hierarchical clustering and admixture analysis based on core genome SNV loci. Admixture here refers to a measure of shared genetic ancestry between isolates. Hierarchical clustering separated the isolates into four clusters corresponding to Groups A, B, C1, and C2, with limited admixture and gene flow between clusters (supplementary fig. 4*A* and *B*, Supplementary Material online). An exception within Group B was a closely related set of four isolates (AZPAE15041, AZPAE14888, AZPAE14878, and BL03) that were estimated to be admixed with ∼40% of their sequences attributable to Group A (supplementary fig. 4*A*, Supplementary Material online, and fig. 1). Interestingly, these four isolates had no clear connections with each other by geographic or clinical isolation source (supplementary table 1, Supplementary Material online). The only other isolate with a similar level of admixture was strain PS75, which in the core genome phylogenetic analysis branched midway between the two major Groups A and B but was distinct from the Group C branch (fig. 1). The provenance of this isolate could not be derived from publicly available information. Although there are other predicted core genome admixture events between isolates within the major clades, admixed isolates represent a small minority of the population. The overall limited amounts of admixture between Group A and Group B isolates supports the possibility that these groups may be independent lineages and consistent with the evolutionary concept of distinct species.

### Identification of Candidate Core Genes That May Be Niche-Adaptive

**Fig. 3.**
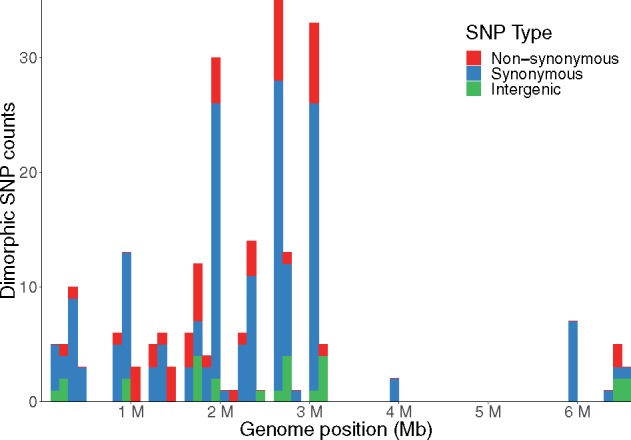
—Dimorphic SNV loci in *Pseudomonas aeruginosa.* Dimorphic SNV loci are defined as core genome positions with one variant in at least 98% of the Group A isolates and a different variant present in at least 98% of the Group B isolates. Each bar represents the total number of dimorphic SNV loci within a 100-kb window relative to the PA14 genome. Numbers of SNVs within coding regions predicted to encode a different amino acid (nonsynonymous mutations) are shown in red, whereas those SNVs not predicted to result in an amino acid change (synonymous mutations) are shown in blue. Numbers of SNVs found within intergenic regions are shown in green.

Fixed differences in core gene loci may point to positive selection in distinct ecological niches. These variations may arise sequentially and become fixed as strains adapt to a new niche or as the result of population-wide gene-specific sweeps mediated by core genome recombination. We examined the core genomes of Group A and B isolates for evidence of group-specific fixed variants. From among 369,282 core genome SNV loci, we identified 240 dimorphic SNV loci with one allele present in at least 98% of Group A isolates and a different allele present in at least 98% of the Group B isolates (supplementary table 2, Supplementary Material online). Interestingly, the group-defining dimorphic nucleotide positions are localized primarily to one half of the *P. aeruginosa* chromosome (fig. 3). Of these 240 dimorphic SNV loci, 213 are located within a total of 89 protein-coding genes; 48 SNVs in 34 genes are predicted to encode nonsynonymous variations. To examine the likelihood that the dimorphic SNV loci may have been identified by chance, each of the isolates in Groups A and B were randomly assigned to either Group A_N_ (541 isolates) or Group B_N_ (186 isolates). The number of SNV loci that were dimorphic in Groups A_N_ and B_N_ were then counted. This analysis was repeated for 1,000 random permutations of isolates into the two groups and in each permutation, 0 dimorphic SNV loci were identified. This indicates that the fixed dimorphic SNVs are unlikely to have occurred by chance.

Assigning putative functional categories to each of the dimorphic-SNV-containing genes using the Clusters of Orthologous Groups of proteins database (Tatusov et al. 1997, 2001) showed that many of the genes containing nonsynonymous differentially fixed mutations were predicted to encode proteins involved in signal transduction (e.g., two-component systems and transcriptional regulators) or metabolic functions (supplementary fig. 5*B* and table 2, Supplementary Material online), perhaps indicating a fine-tuning of signaling and metabolic capacities to meet the requirements of different niches. Evaluation of fixed versus polymorphic variants using the McDonald–Kreitman test (McDonald and Kreitman 1991) revealed that several of the 34 genes with dimorphic SNVs predicted to encode nonsynonymous variants had neutrality index values below 1, suggesting positive selective pressure (Rand and Kann 1996). However, none of the differences were statistically significant (supplementary table 3, Supplementary Material online). The lack of significance is likely secondary to the low numbers of variants causing reduced statistical power, but we cannot exclude the possibility that substantial selective pressures may not differ within versus between groups at the level of individual genes. Despite this, the presence and characteristics of genetic loci containing dimorphic SNPs could suggest a trend toward fixation of particular variants within the groups, potentially reflecting adaptation of Group A and B isolates to their respective ecological niches.

**Table 2:** Core, Accessory, and Pangenome Characteristics

	Size (bp)	% GC
Core genome^a^	5,784,306	66.94
Accessory genome^b^	911,794 (276,874–2,193,688)	61.58 (59.1–65.48)
Unique accessory genome	26,280,940	57.19
Pangenome	32,065,171	58.98

^a^95% core genome, that is, sequence present in ≧702 of 739 isolates.

^b^Values are medians. Values in parentheses are minimum and maximum values.

### Accessory Genome Differences Support a Barrier to Genetic Exchange between Group A and Group B Isolates

**Fig. 4.**
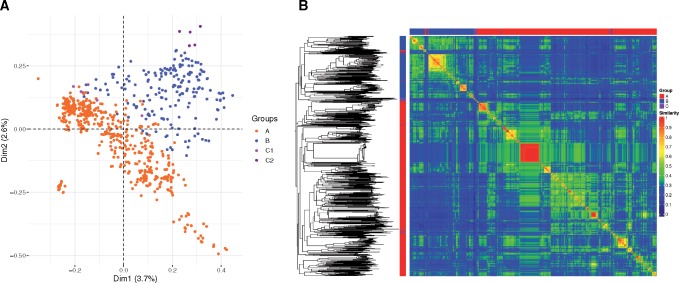
—Accessory genome content of Group A and Group B isolates. (*A*) Multiple correspondence analysis of AGEs at least 100 bp in length. Orange = Group A, blue = Group B, pink = Group C1, and purple = Group C2. (*B*) A neighbor joining tree was generated from the Bray–Curtis distances calculated from AGEs at least 100 bp in length and midpoint rooted. The major group memberships of isolates are indicated in the columns along the left and upper axes of the heatmap (red = Group A, blue = Group B, and purple = Group C). The heatmap shows pairwise accessory genome content similarities based on inverse Bray–Curtis distances (1 – *d*) according to the scale bar.

Similar to core genome recombination, horizontal transfer of AGEs may also be limited in strains inhabiting distinct ecological niches. For this reason, we next examined the distribution of AGEs in the two large groups of *P. aeruginosa*. Characteristics of the core-, accessory-, and pangenomes of the sequence collection are shown in table 2. Analysis of the pangenome and novel sequences identified in each additional genome suggests that, similar to the population as a whole, the pangenome of *P. aeruginosa* Groups A and B are open (supplementary fig. 6, Supplementary Material online) (Tettelin et al. 2008). A total of 7,239 unique contiguous AGE sequences at least 200 bp in length were identified; these were further subdivided into 68,830 discrete AGE subelements. Multiple correspondence analysis of the 21,453 AGE subelements at least 100 bp in length showed that the accessory genomes of Group A, B, C1, and C2 isolates are relatively distinct (fig. 4*A*). Bray–Curtis distances based on presence or absence in each isolate of discrete accessory sequences at least 100 bp in length were calculated, used to produce a neighbor joining tree, and visualized as a heat map of isolate-isolate accessory genome content similarity (fig. 4*B*). This analysis showed that the accessory genomes of Group A isolates were overall more similar to each other than to those of Group B isolates, and vice versa. Similarly, an analysis of the pangenome sizes of 1,000 random subsets of genomes from each group showed that the average pangenome size of isolates from Groups A and B together was significantly larger than the average pangenome size of isolates within either Group A or Group B alone (supplementary fig. 7, Supplementary Material online). Together, these results suggest that Group A and Group B isolates have acquired a somewhat different albeit overlapping set of accessory genome sequences.

We next examined the distribution of previously characterized GIs in the *P. aeruginosa* groups. As GI sequences can be highly mosaic and fragmentary between isolates, we focused on subsets of these GIs. We used validated PCR primer sequences and an in silico “PCR amplification” approach to detect portions of the GIs PAGI-1, PAGI-2, PAGI-3, PAGI-4, PAPI-1, PAPI-2, and pKLC102 (Morales-Espinosa et al. 2012). Consistent with the Bray–Curtis analysis, most GI sequences were identified in members of both Group A and Group B, although portions of PAGI-4, PAPI-1, PAPI-2, and pKLC102 were found to have a statistically significant overabundance in one group or the other (supplementary table 4, Supplementary Material online). These results demonstrate that many of the characterized *P. aeruginosa* GIs are found in isolates from both Groups A and B but that some are not evenly distributed between the groups. This may indicate that some GIs are preferentially lost or gained in one ecological niche or the other and/or that genetic barriers exist such that some GIs are more easily transferred between isolates within a group than across groups.

**Fig. 5.**
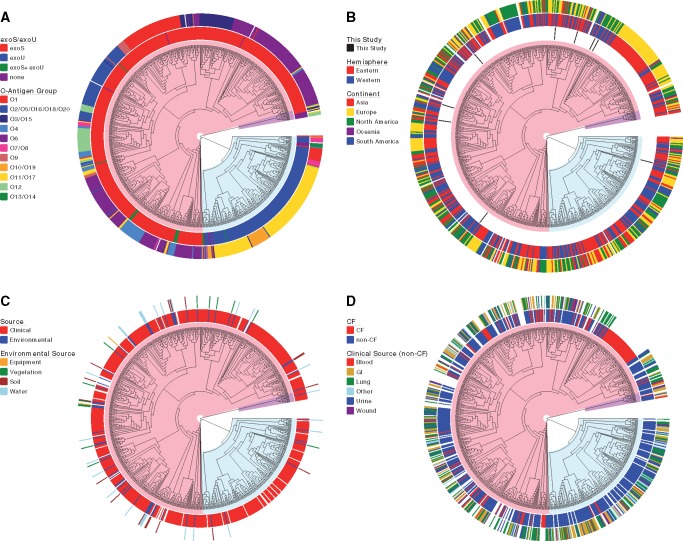
—Isolate demographic and accessory genome characteristics. Each panel shows 95% core genome maximum likelihood trees with isolate information highlighted. Trees are displayed as phylograms with branch lengths that do not correspond to genetic distances. Major clonal groups are highlighted in red (Group A), blue (Group B), and purple (Group C). (*A*) Accessory genome characteristics. Inner ring: Presence of type III effector genes *exoS* and *exoU*. Outer ring: O-antigen biosynthesis locus type. (*B*) Geographic source of isolates. Inner ring: isolates sequenced as part of this study. Middle ring: global hemisphere of isolation. Outer ring: continent of isolation. (*C*) Source of environmental isolates. Inner ring: environmental versus clinical isolates. Outer ring: specific sources of environmental isolates. (*D*) Source of clinical isolates. Inner ring: CF versus non-CF clinical isolates. Outer ring: body site of isolation for the non-CF isolates.

O-antigen polysaccharides, which comprise the terminal portion of lipopolysaccharide (Rocchetta et al. 1999), are common receptors for phages that infect *P. aeruginosa* and therefore are under strong selection (Temple et al. 1986). Although nearly every isolate of *P. aeruginosa* has an O-antigen biosynthesis island at the same genomic locus, these islands vary significantly in the number and types of genes they carry (Raymond et al. 2002; Kung et al. 2010). We therefore examined the genes at the O-antigen biosynthetic locus in each isolate. Many of the O-antigen biosynthesis islands differed significantly in their incidence in one phylogenetic group relative to the other (fig. 5*A* and supplementary table 5, Supplementary Material online). In particular, the predominant O-antigen biosynthetic island type, O6, was found exclusively in Group A isolates, whereas the O11 island predominated in Group B isolates. These findings suggest that different O-antigen types may provide differential selection in distinct niches inhabited by Group A and Group B isolates.

### Identification of Candidate Accessory Genes That May Provide Niche-Adaptive Characteristics

Bacteria can adapt to new niches following horizontal gene transfer of an adaptive gene or genes (Cohan and Koeppel 2008). However, genes adaptive for one niche may confer a cost when transferred into another niche and can thus be recognized by their nearly universal presence in isolates from one niche but not the other (Cohan 1994). Our examination of characterized GIs indicated that portions of some islands were overrepresented in one group or the other (supplementary table 4, Supplementary Material online). To further investigate this in an unbiased manner, we applied filters to detect all AGEs found in at least 90% of isolates in Group A and no more than 10% of isolates in Group B, and vice versa. From a total of all 68,830 AGEs at least 1 bp in length, 11 contiguous groups of AGEs were identified as being predominantly found in Group A isolates. These 11 AGE groups contained portions of 8 different genes, including genes predicted to encode a pilus assembly chaperone and components of an ABC transporter (supplementary table 6, Supplementary Material online). A total of 26 complete or partial genes in 16 AGE groups were found predominantly in Group B isolates. These included genes predicted to encode a protein disulfide isomerase, a potassium uptake protein, a nucleoside-binding outer membrane protein, and a zinc-binding oxidoreductase (supplementary table 6, Supplementary Material online). As mentioned, these genes could potentially play a role in allowing *P. aeruginosa* to better persist is specific environmental niches. In this regard, it is interesting that one of the AGEs predominant in Group B isolates, PAgrpB_7, consists of the GI RGP32, which had been previously described to contain stress-associated genes such as the flavodoxin-encoding gene *fldP* (Moyano et al. 2014). The cyanobacterial flavodoxin in this island has been shown to promote *P. aeruginosa* survival in mammalian macrophages and increase virulence in *Drosophila* infections.

Analyses of 1,000 random reshufflings of the isolates into two groups containing 541 and 186 isolates did not identify any AGEs that were similarly predominant in one or the other random group. Similarly, no group-predominant AGEs were found following 1,000 random reshufflings of isolates into two groups containing balanced numbers of 360 isolates each.

These results identify several group-associated accessory genes. Although it is possible that one or more of these genes provides a niche-specific selective advantage to isolates in that Group, the nonrandom association of these genes with isolates in a particular Group could also be the result of early acquisition and subsequent propagation after niche specialization independent of any specific evolutionary advantage or barriers to gene flow between them.

The preceding analysis also identified the *exoS* and *exoU* genes as being highly segregated between Groups A and B. These genes encode effector proteins of the *P. aeruginosa* type III secretion system. It has been previously reported that nearly every isolate of *P. aeruginosa* has either the *exoS* gene or the *exoU* gene, with only rare isolates containing both genes or neither gene (Feltman et al. 2001; Lomholt et al. 2001; Garey et al. 2008; Pirnay et al. 2009; Bradbury et al. 2010). Of note, 528 (98%) of the 541 Group A isolates contained *exoS* but not *exoU*, and 176 (95%) of 186 Group B isolates contained *exoU* but not *exoS* (fig. 5*A* and supplementary table 5, Supplementary Material online). Performing in silico PCR using primer sequences against the middle portion of the *exoU* gene (bases 1098–1531) yielded identical findings as those shown for the full *exoU* gene in supplementary table 5, Supplementary Material online. These results indicate *exoS* and *exoU* discriminate Group A and Group B isolates with a high degree of accuracy and suggests the genes could provide a fitness advantage in the respective ecological niches they inhabit.

We next examined the genetic context in which *exoU* and *exoS* occurred. As the *exoU* gene is present in the GI PAPI-2 (He et al. 2004; Morales-Espinosa et al. 2012), we examined the distribution of other portions of PAPI-2 in the isolates. Of the other two portions of PAPI-2 evaluated with primer sets, one (RS07–RS08) showed a statistically significant overabundance in Group B (86.0%) compared with Group A (46.6%) but this was not to the same degree as the *exoU* gene (95.2% vs. 1.8%) (supplementary table 4, Supplementary Material online). The other portion of PAPI-2 screened (*xerC*) was equally distributed between isolates in the two groups. Thus, both Group A and Group B isolates contained portions of PAPI-2, but the *exoU* gene itself was largely restricted to Group B isolates. Of the ten *exoS+* Group A isolates that also contained *exoU* (supplementary table 1, Supplementary Material online), nine had *exoU* and its chaperone gene *spcU* located immediately upstream (2,064 and 414 bp, respectively) of conserved core gene PA0988, the same location in which they are found in the Group B strain PA14. In the tenth isolate (ATCC 25324), the *exoU* and *spcU* genes could not be definitively localized in the chromosome due to their presence on extremely short contigs in the assembly. We next examined the location of the *exoS* gene by performing in silico PCR using primers spanning the two genes immediately flanking the *exoS* gene (the *exoS* chaperone gene *spcS* and the hypothetical protein gene PA3840). In all Group A isolates and in the single Group B isolate that contained the *exoS* gene (AZPAE14404), it was found in this context. In all other Group B isolates, both flanking genes were present but the entire *exoS* gene was absent. The presence of the adjacent *exoS* chaperone gene in all *P. aeruginosa* strains of both groups supports prior hypotheses that the *exoS* gene predated acquisition of *exoU* in *P. aeruginosa* and was lost due to a targeted deletion event (Kulasekara et al. 2006).

Interestingly, several of the ten *exoU+/exoS+* isolates in Group A were phylogenetically distinct from each other (fig. 5*A*). These findings are consistent with either the rare acquisition of *exoU* by a few Group A isolates or the general loss of *exoU* from nearly all Group A isolates. As mentioned above, a closely related set of four Group B isolates (AZPAE15041, AZPAE14888, AZPAE14878, and BL03) were admixed with ∼40% of their sequences attributable to Group A. Interestingly, these four isolates lacked both the *exoS* and *exoU* genes (fig. 5*A*). These were also the only four isolates in the population that had O13/O14-type O-antigen biosynthesis loci (fig. 5*A*). These isolates may represent a lineage evolving from an ancestor that either lost or failed to acquire the *exoU* gene, perhaps altering their niche specificity and again providing opportunities for recombination with *exoS+* isolates.

### Intergroup Nucleotide Identity Varies More than Intragroup Nucleotide Identity

The preceding results suggest that Group A and Group B isolates represent two lineages but it is unclear how distinct these lineages are (Wiley 1981). Although the criteria for defining bacterial species are evolving (Doolittle and Papke 2006; Krause and Whitaker 2015), one proposed metric is average nucleotide identity (ANI) of genome sequence pairs. A cutoff of 95–96% ANI was found to correspond to a 70% DNA–DNA hybridization threshold traditionally used for species delineation (Goris et al. 2007; Richter and Rosselló-Móra 2009). To examine the nucleotide relatedness of Group A and Group B isolates, the ANI values of the 739 isolates were calculated for every combination of pairs of genome sequences and their reciprocals. This showed that the average ANI among all Group A and all Group B isolates was 99.3% and 99.2%, respectively, and that the ANI of isolates between Groups A and B was slightly lower at 98.76% (table 3). Group C1 ANI values against Group A or Group B isolates were lower still at 98.2% and 98.0%, respectively. Group C2 isolates, representing the PA7-like outlier isolates, shared just 93.49% ANI with Group A isolates, 93.51% ANI with Group B isolates, and 93.38% ANI with Group C1 isolates. These results suggest that Groups A, B, and C1 would not be considered separate species by commonly accepted ANI criteria, although the evolutionary species concept suggests they are independent lineages. The classification of PA7-like Group C2 isolates as belonging to the same species as the other groups in *P. aeruginosa* may warrant further discussion.

**Table 3:** Average Nucleotide Identities (ANIs)

Average (Standard Deviation)						
	Group A	Group B	Group C1	Group C2	CF-PA39|JDVE	PS75|JIEP
Group A	99.31 (0.1484)					
Group B	98.76 (0.1206)	99.15 (0.2707)				
Gropu C1	98.20 (0.0648)	98.03 (0.0598)	99.42 (0.2248)			
Group C2	93.49 (0.1081)	93.51 (0.1009)	93.38 (0.1075)	99.03 (0.2409)		
CF-PA39|JDVE	97.54 (0.0480)	97.46 (0.0510)	97.18 (0.0318)	93.40 (0.0774)	NA (NA)	
PS75|JIEP	99.08 (0.0485)	99.03 (0.0620)	98.14 (0.0330)	93.49 (0.0849)	97.47 (0.0063)	NA (NA)

### Group A and Group B Isolates Are Associated with Somewhat Different Demographic Characteristics

As mentioned, the separation of most *P. aeruginosa* isolates into one of two large phylogenetic groups suggests the possibility that these two populations may inhabit two different niches (Cohan 2002b). To support this conjecture, we examined the sources of the isolates. Isolates did not group based on continental and hemispheric origin (fig. 5*B* and supplementary table 5, Supplementary Material online). Although isolates of clinical or environmental origin were found in both major branches of the tree, a significantly greater proportion of environmental isolates was observed in Group A than in Group B (*P* < 0.01; fig. 5*C* and supplementary table 5, Supplementary Material online). Furthermore, differentiation of environmental isolates by specific source (e.g., equipment, vegetation, soil, and water) showed no significant predominance of any sources in one group over the other (fig. 5*C* and supplementary table 5, Supplementary Material online). For isolates identified as originating from clinical sources, we first separated isolates into “CF” and other clinical sources (“non-CF”). We made this distinction because some reports have suggested that *P. aeruginosa* isolates from patients with CF are phenotypically and genotypically distinct from other *P. aeruginosa* isolates (Tummler et al. 1997; Oliver et al. 2000; van Mansfeld et al. 2010). Although non-CF clinical isolates were found in both major branches of the phylogenetic tree, all but 3 of the 115 isolates cultured from CF patients were in Group A (fig. 5*D* and supplementary table 5, Supplementary Material online). This statistically significant predominance (*P* < 1 × 10^−10^) of CF isolates in Group A was maintained even when all 48 isolates belonging to the Liverpool Epidemic Strain clonal group, a CF epidemic strain (Scott and Pitt 2004), were removed (supplementary table 7, Supplementary Material online). Among the non-CF clinical isolates, those cultured from eye, ear, or nose sources were predominantly Group B (fig. 5*D* and supplementary table 5, Supplementary Material online). In this group of isolates, the majority (34 of 40) had been cultured from eye infections (supplementary table 1, Supplementary Material online). Isolates from other clinical sources were more evenly distributed between Groups A and B. These findings suggest that CF patients may be more likely to acquire their *P. aeruginosa* isolates from reservoirs of Group A isolates than Group B isolates. Alternatively, Group B isolates may be less fit to colonize and infect the airways of CF patients than Group A isolates. Eye infections represent an inverse situation in which isolates are more likely acquired from Group B reservoirs or in which Group B isolates are better able to cause these infections.

## Discussion

We used the whole-genome sequences of 739 *P. aeruginosa* isolates to confirm previous reports that the population structure of *P. aeruginosa* consists of two large clades and one or more smaller clades (Stewart et al. 2011; Freschi et al. 2015, 2018, 2019; Hilker et al. 2015; Marvig et al. 2015; Williams et al. 2015). Despite earlier observations of this population structure, the underlying reasons for this distinct segregation in *P. aeruginosa* have not previously been extensively explored. One explanation for the striking separation of the two large clades comprising Groups A and B is that the bacteria in these groups inhabit distinct ecological niches. Consistent with this notion is that several clusters of core genome SNPs are characteristic of Group A or Group B. Similar, although less marked, differences between the two major groups were also seen in the accessory genome. This analysis identified several genes that may be contributing to the ability of bacteria in each group to better persist in different ecological niches, the most noteworthy being *exoS* and *exoU.* As the two groups diverged through adaptation to different niches, barriers would have progressively limited intergroup but not intragroup genetic exchange. Evidence of decreased intragroup recombination was indeed observed between Group A and Group B isolates indicating they are independent lineages that fit an evolutionary species concept. Although predominantly *exoS+* Group A strains and predominantly *exoU+* Group B strains are found to be phylogenetically divergent, the genetic differences between the two groups did not meet ANI criteria for distinct species. The same, however, cannot be said of the Group C2 clade; although ANI values are not sufficient on their own to delineate species, these isolates are quite distinct, and future studies should focus on whether they should remain within the *P. aeruginosa* species.

Genetic isolation is evidenced by relatively little gene flow between Group A and Group B in the core genome. We observed that just 14–19% of core genome recombination events could be attributed to sources outside of each major group (fig. 2). Previous studies have found that *P. aeruginosa* is characterized by a low overall recombination rate within the core genome—only one-fifth the rate of mutation (Dettman et al. 2015)—but it has also been shown through the distribution of syntenic SNPs that free recombination occurs between the core genomes of major clones (Hilker et al. 2015). Consistent with our findings, it has been reported that the characteristics of syntenic SNP haplotypes varied depending on whether interclonal or intraclonal isolate pairs were compared (Losada and Tummler 2016). Our results suggest that the groups have diverged to the extent that sequence differences hinder homologous recombination, that genetic barriers to recombination (such as restriction-modification systems) exist between the two groups, or that distinct ecological niches provide a physical barrier to gene transfer.

Our finding that patterns of accessory genome content of isolates within groups are overall more similar than between groups is further evidence for differentiation between Groups A and B. A study of regions of genomic plasticity (RGPs) among 40 *P. aeruginosa* isolates also demonstrated distinct accessory genome compositions between the two major groups (Freschi et al. 2018). Much of the accessory genome of *P. aeruginosa* is composed of horizontally transferred elements acquired from environmental reservoirs (Kung et al. 2010), and differences in accessory genome content suggest exposure to distinct reservoirs. A second possible interpretation is that genetic barriers limit efficient horizontal transfer of specific accessory elements into one group but not the other or between groups.

Our analysis identified a number of core gene alleles and accessory genes that are discriminatory for Group A and B isolates. Because these genes and alleles are relatively exclusive to one group or the other, they are candidates for niche-adaptive genes, although McDonald–Kreitman testing did not show statistically significant evidence of positive selection. Arguably, the most interesting of the group-discriminatory accessory genes are *exoS* and *exoU.* That these genes could be niche-adaptive has been suggested (Wolfgang et al. 2003; Pirnay et al. 2009). Previous studies have also suggested a phylogenetic separation between *P. aeruginosa* isolates containing these two different type III effector genes (Wiehlmann et al. 2007; Selezska et al. 2012), which is confirmed by our study. Previous reports have shown that isolates with these type III effector genes are associated with infections of different character and severity (Finck-Barbançon et al. 1997; Hauser et al. 1998, 2002; Schulert et al. 2003; Shaver and Hauser 2004; El-Solh et al. 2012; Pena et al. 2015). The strong association of these genes with separate phylogenetic groups combined with our findings that very few other accessory genes are similarly group-exclusive raises suspicion that these genes may play an important role in niche adaptation and/or establishing a genetic barrier between the groups.

The genetic mechanisms that account for the separation of the *exoS* and *exoU* genes into Groups A and B are unknown. The *exoU* gene is thought to have been acquired by horizontal gene transfer into Group B isolates, as it is located within a highly variable GI inserted into a chromosomal tRNA^Lys^ gene (Kulasekara et al. 2006). The provenance of *exoS* is less clear. This gene may have been present in an early ancestor of all *P. aeruginosa* strains and subsequently lost from Group B isolates, or Group A isolates may have acquired *exoS* by horizontal gene transfer early in this group’s divergence from Group B. Some evidence supports the former hypothesis. The nucleotide sequence of *exoS* is 80.2% identical to the effector gene *exoT*, which is present in all Group A and Group B isolates, suggesting that *exoS* and *exoT* arose very early from a duplication event (Yahr et al. 1996). The *spcS* chaperone gene, which is immediately adjacent to *exoS* in Group A isolates, is found in both Group A and Group B isolates, again consistent with deletion of *exoS* in Group B isolates. Likewise, sequencing studies suggested that *exoS* has been deleted from Group C2 PA7-like strains (Huber et al. 2016), so there is a precedent for loss of *exoS* from a group of isolates. Deletion of *exoS* is postulated to have occurred through a recombination event involving inverted repeats bordering the gene and that this targeted deletion was caused by an *exoU-*linked gene at the time of *exoU* acquisition (Kulasekara et al. 2006).

In addition to *exoS* and *exoU*, several other genes were highly associated with Group A or Group B and are candidates for niche-adaptive genes. One prominent example is the GI RGP32 in Group B strains. The stress response genes in this island, including a flavodoxin gene with a demonstrated immunoprotective function (Moyano et al. 2014), may contribute to survival of these strains in eukaryotic hosts. Other accessory genes that were highly associated with one group or the other tended to encode for hypothetical proteins or had undefined functions. Hence, it is not clear how they might contribute to niche adaptation. The prominence of fixed dimorphic variants within core genes with purported signal transduction mechanisms suggests they may play important roles in specialization to particular environments. Relative and absolute preponderances of certain O-antigen biosynthesis loci among isolates of one or the other groups suggest that these loci became fixed after differentiation and/or that the O-antigen biosynthesis locus may contribute to niche specialization. An important consequence of these findings is that phenotypic differences between isolates in Groups A and B that were previously attributed to a single gene (e.g., virulence caused by *exoS* or *exoU*) may have in fact been due to the cumulative effects of multiple group-discriminatory genes and core genome alleles (Schulert et al. 2003; Pena et al. 2015). Additional studies may uncover interesting roles in pathogenesis for these group-discriminatory genes and alleles.

The observation that *P. aeruginosa* has a population structure consisting of distinct groups led us to ask whether these groups had diverged to the extent that they may represent distinct species. Although criteria for species designation are controversial, a number of groups have suggested that ANI is useful in this regard. Consistent with previous analyses of intraspecies sequence diversity in *P. aeruginosa* (Hilker et al. 2015), we found that Group A, B, and C1 *P. aeruginosa* isolates had intergroup ANI values of >98%, which supports inclusion within a single species. The fact that these three lineages appear to be evolutionarily independent with low intergroup recombination suggests that current species definitions based on ANI may indeed be broader than those based on evolutionary species concepts (Wiley 1978). In contrast, Group C2 isolates had intergroup ANI values <94%, which falls outside the traditional species threshold by ANI. Whereas most Group A and Group C1 isolates contain *exoS* and most Group B isolates contain *exoU*, the Group C2 isolates, such as PA7, have neither gene. Furthermore, they lack the genes encoding the type III secretion apparatus (Roy et al. 2010) and instead have acquired a type-V-secreted toxin, exolysin (Elsen et al. 2014). Together, these results suggest that if the cause of genetic isolation in *P. aeruginosa* is ecological, isolate groups could potentially inhabit distinct ecological niches. Based on these findings, further studies should be considered to characterize the taxonomic classification PA7-like Group C2 bacteria.

Our findings suggest that Group A and Group B isolates may be associated with distinct ecological niches, so we sought to determine what these niches might be. We found that isolates in each major clade were distributed globally across both the Eastern and Western hemispheres as well as among continents, so geographic separation did not account for segregation into these groups. As was also noted by Wiehlmann et al. (2015), we found that major groups of *P. aeruginosa* were cultured from both environmental and clinical sources. However, relatively fewer Group B isolates were from the natural environment, suggesting that these isolates may be more adapted to healthcare settings, human hosts, or nonnatural settings. This was particularly apparent in isolates from eyes, ears, and noses of patients and agrees with prior reports of *exoU+* isolates being common in infections of these sites (Lomholt et al. 2001; Stewart et al. 2011; Rutherford et al. 2018). Isolates from individuals with CF were an exception and were rarely members of Group B. The previously reported predominance of *exoS*+ isolates among *P. aeruginosa*-infected CF patients is consistent with this finding (Feltman et al. 2001), although it is unlikely that the CF lung environment itself is driving adaptation of Group A isolates. With the exception of specific epidemic strains (e.g., Liverpool Epidemic Strain), it is currently believed that most CF isolates are not transmitted back to the environment or to another individual with CF (Parkins et al. 2018). One explanation for these findings is that *P. aeruginosa* inhabits geographically overlapping but distinct microenvironments, and that patients with different types of infections acquire their *P. aeruginosa* isolates from different environmental reservoirs*.* In one scenario, the group-discriminatory genes could provide defense against different predators found in the distinct environmental niches. Indeed, the *exoS* and *exoU* genes allow *P. aeruginosa* to kill amebae (Abd et al. 2008; Matz et al. 2008). Likewise, in other bacterial species, amebae recognize O-antigen types with differing efficiencies, which may drive selection of different O-antigen types in specific environments (Wildschutte et al. 2004; Atzinger et al. 2016). Thus, the *exoS/exoU* genotypes and O-antigen serotypes of Group A and B isolates may vary because these isolates inhabit different ecological niches, each with its own distinct set of amebae or other predators. Another possibility is that most patients are exposed to both Group A and Group B *P. aeruginosa* isolates but that the genes specific to each group favor the establishments of different types of infections. These hypotheses need to be further explored with a larger number of isolates from diverse sources.

Our study has some important limitations. First, this study cannot definitively determine whether the genetic isolation between Group A and Group B resulted from ecological or biological factors. Further studies, perhaps with more detailed geographic and environmental metadata for isolates, will be required to address this question. Second, the *P. aeruginosa* genomes in NCBI are not a random collection, and some sets of isolates are overrepresented as the result of sequencing of multiple very similar isolates, whereas isolates from other sources are underrepresented or absent. For example, relatively few isolates from nonclinical sources were available in the NCBI database when this study began. We were able to supplement these numbers somewhat by sequencing nine more environmental isolates, but substantially more environmental isolates should be included in future studies. Another potential limitation is that the genomes in the database were provided by multiple contributors to a public database and varied in quality of both sequencing and assembly. Hence some genes and/or genomic regions may have been omitted from lower quality assemblies. Nevertheless, in only 11 of the 739 genome sequences (1.5%) could neither the *exoU* nor the *exoS* gene be identified, which is consistent with the prevalence of *exoS*-/*exoU*-isolates in other reports (Berthelot et al. 2003; Pirnay et al. 2009). Finally, we found few Group C1 and C2 isolates, which precluded a more thorough analysis of these groups. It is unclear whether isolates in these groups are truly rare relative to Group A and Group B isolates, or whether they were underrepresented due to sampling bias. As the number of *P. aeruginosa* isolates sequenced and deposited in public databases continues to grow, future studies may more fully define characteristics of isolates within these groups, their relationships to the species population structure, and the drivers of genetic isolation in *P. aeruginosa*.

## Conclusions

We used a large collection of *P. aeruginosa* whole-genome sequences to confirm that the majority of isolates segregated into two distinct groups. In addition to the phylogenetic distance between the groups, infrequent intergroup recombination relative to intragroup recombination and greater intragroup accessory genome similarity suggests that they are genetically isolated. A small set of core genome alleles and accessory genes discriminated between these two groups. This set included *exoS* and *exoU* (type III secretion effector genes) and RGP32, which encodes a flavodoxin gene implicated in virulence, among others. These genes and alleles are candidates for niche-adaptive factors. Although genetic differences between Groups A, B, and C1 did not meet standard ANI criteria for categorization as separate species, Group C2 isolates warrant further consideration for reclassification. Further studies are necessary to determine whether ecological and biological barriers separate these three groups, the specific ecological niches occupied by different *P. aeruginosa* groups, and how genetic differences contribute to the adaptation of each group.

## Supplementary Material

Supplementary data are available at *Genome Biology and Evolution* online.

## Supplementary Material

Supplementary_Material_evz119

## Notes

**Data deposition:** All genomic sequences are deposited at NCBI (www.ncbi.nlm.nih.gov). Accession numbers are provided in the Materials and Methods section and supplementary materials, Supplementary Material online.
