# Supplementary material for: The Population Structure of Pseudomonas aeruginosa Is Characterized by Genetic Isolation of exoU+ and exoS+ Lineages
Source: Genome Biol Evol. 2019 Jun 7;11(7):1780–96. doi: 10.1093/gbe/evz119 (PMC6690169; doi:10.1093/gbe/evz119)

Supplemental Figure Legends

Supplemental Figure 1. Population structure of *P. aeruginosa* isolates using a reference-based SNP-calling approach. A maximum-likelihood phylogenetic tree was generated based on 95% core genome SNP loci using whole-genome alignment to a reference genome sequence (PA14) and variant calling with the MUMmer software package. Leaves are colored based on their respective group assignments in the kSNP-derived tree shown in Figure 1: Group A (red), Group B (green), and Group C (purple). The scale bar represents genetic distance.

Supplemental Figure 2. Population structure of *P. aeruginosa* isolates corrected for recombination. The reference-based core genome phylogenetic tree is shown with branch lengths corrected by ClonalFrameML to remove the influence of recombination. Leaves are highlighted with colors based on the kSNP analysis shown in Figure 1: Group A (red), Group B (green), and Group C (purple). The scale bar represents genetic distance. Note the difference in scale between this figure and Figure 1.

Supplemental Figure 3. Recombination parameters of isolate subsets. Subsets of 100 isolate sequences were randomly selected from among either Group A (red) or Group B (blue) isolates. ClonalFrameML recombination analysis was performed on each subset of sequences. Random selection and analysis were performed 10 times for each Group. In each boxplot, the median value of the 10 repeat analyses is represented by the thick horizontal bar, first and third quartiles are represented by the bottom and top of the box, respectively, and whiskers represent 1.5 x the interquartile range. Outliers are

represented by “X”. The mean of the 10 repeat analyses is represented by the open diamond. Values obtained from analysis of the complete sequence set as given in Table 1 are represented by open circles. (A) Values of R/theta, or relative rate of recombination to mutation. (B) Values of delta, or the mean DNA import length. (C) Values of nu, or the mean divergence of imported DNA sequences. (D). Values of r/m, or the relative contributions of recombination vs. mutation to diversity.

Supplemental Figure 4. Core genome admixture analysis. The tree is shown as a phylogram in which branch lengths do not correspond to genetic distances. Major clonal groups are highlighted in red, blue, and purple corresponding to the groups indicated in Figure 1. (A) Per-isolate core genome relative admixture amounts as calculated by BAPS are indicated in the ring surrounding the tree. The labels indicate the names of some of the most admixed isolates. (B) A gene flow diagram is shown with arrows indicating the direction of gene flow from source clusters to target clusters. The vector labels represent the relative amounts of ancestry in the source clusters among individuals assigned to the target clusters.

Supplemental Figure 5. Predicted functional categories of genes containing dimorphic SNVs. Numbers of genes in each (A) COG category and (B) COG subcategory are shown. Blue portions of bars represent numbers of genes in each category with only synonymous SNVs. Orange portions of bars represent numbers of genes in each category with one or more non-synonymous SNVs.

Supplemental Figure 6. Pangenome and new genome sizes. (A) Average pangenome sequence size of isolates from Group A only (purple), isolates from Group B only (orange), isolates from Group A and Group B (purple), and the full population (blue). Circles represent the average pangenome size from 1000 random permutations of N genomes. Dashed lines represent the best-fit line resulting from fitting the data to a power law function ($y = b * x^{a}$). (B) Average new genome size for isolates from each of the groups and population. Circles represent the average amount of new genome sequence found in the last genome from 10,000 random permutations of N genomes. Dashed lines represent the best-fit line resulting from fitting the data to power law function ($y = b * x^{-a}$). (C) Parameters of least squares fit of the power law ($y = b * x^{-a}$) for average new genome values shown in Supplemental Figure 5B. R-squared values shown are for linear regression of actual values vs predicted values. Values of 'a' parameter for each group and combination of groups are less than 1 suggesting the pangenomes of the species, as well as isolates in each group, are open.

Supplemental Figure 7. Relative pangenome size of 100 random genome sequences by Group. Using the output of the Spine analysis of the 739 isolate genomes, pangenome sequence size was calculated for 1000 random permutations of 100 genome sequences from isolates in Group A ("A"), isolates in Group B ("B"), isolates in Groups A and B ("A+B"), and isolates from the total set ("A+B+C"). In each boxplot, the median pangenome size of the 1000 repeat analyses in each set is represented by the thick horizontal bar, first and third quartiles are represented by the bottom and top of the box, respectively, and whiskers represent 1.5 x the interquartile range. Outliers are

represented by solid dots. The mean of the 1000 repeat analyses is represented by the open diamond. Bars with double asterixis represent comparisons of the means of two sets by Student's t-test with p-values < 0.001.

Supplemental Figure 1

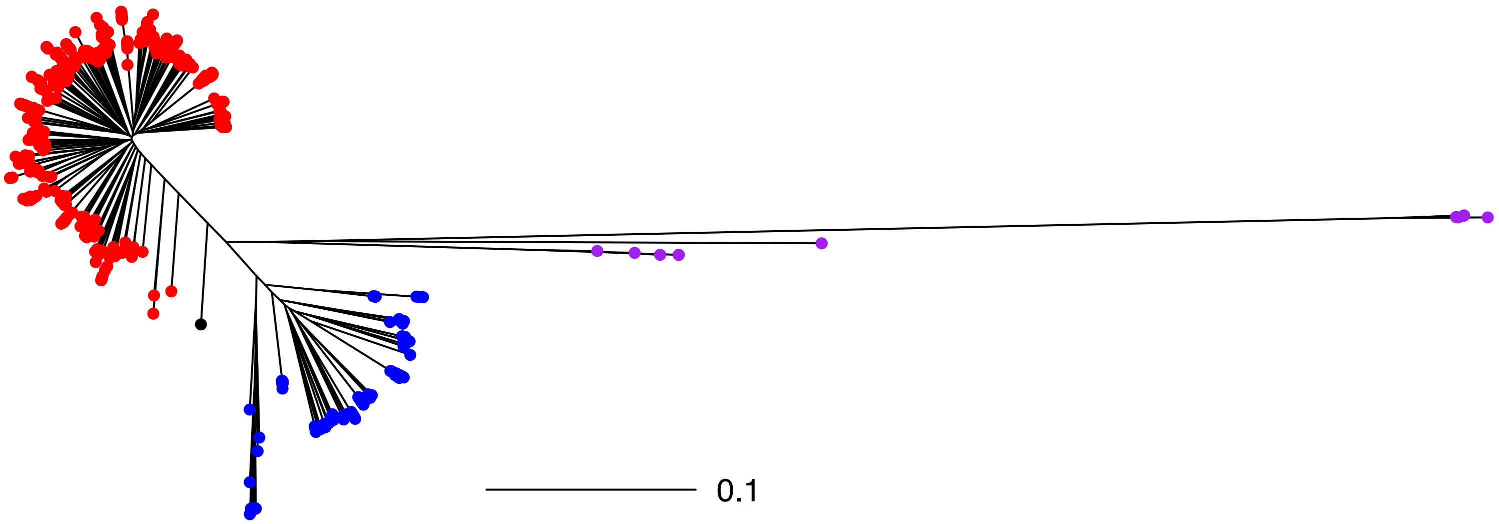

Supplemental Figure 2

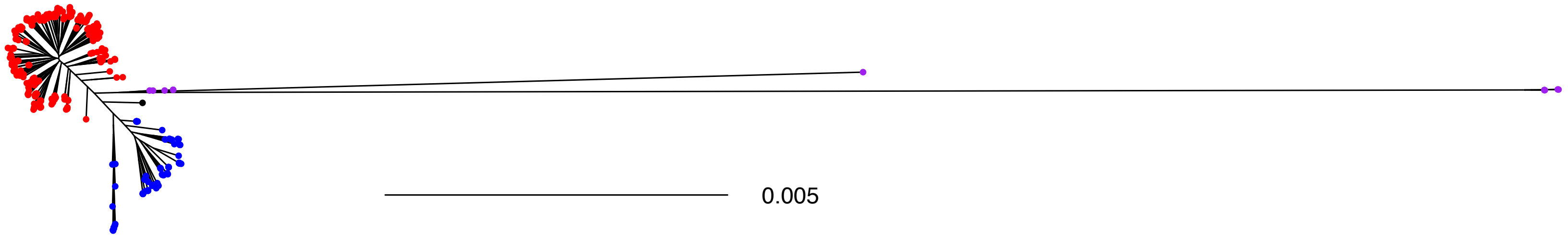

Supplemental Figure 3

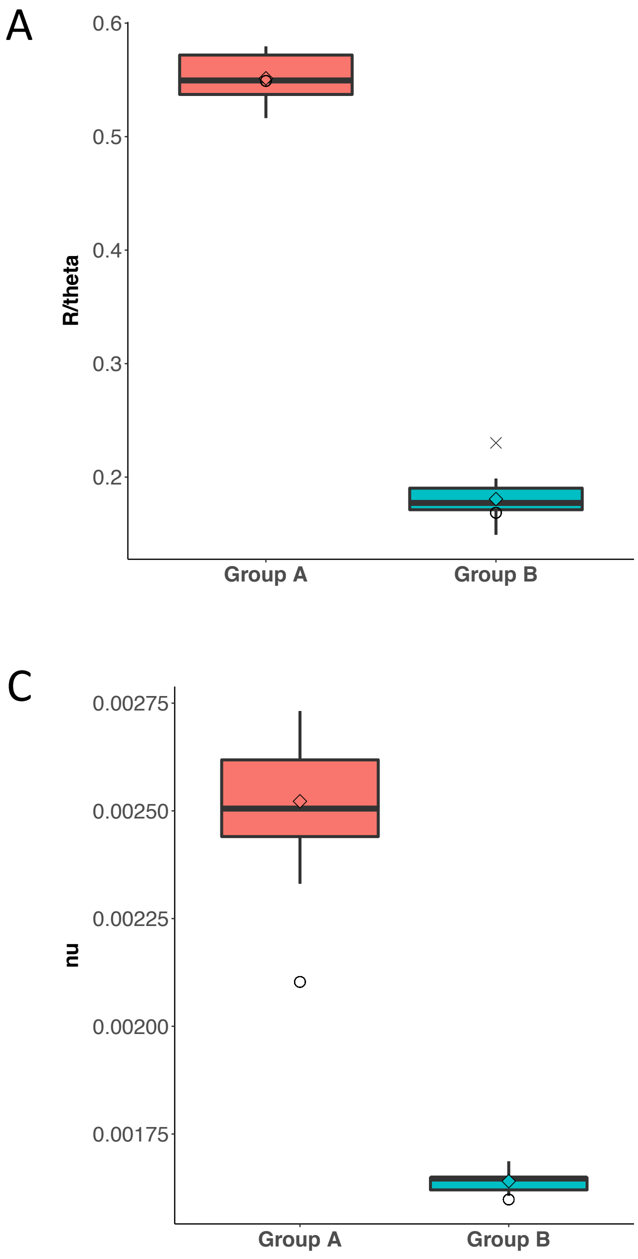

Supplemental Figure 4

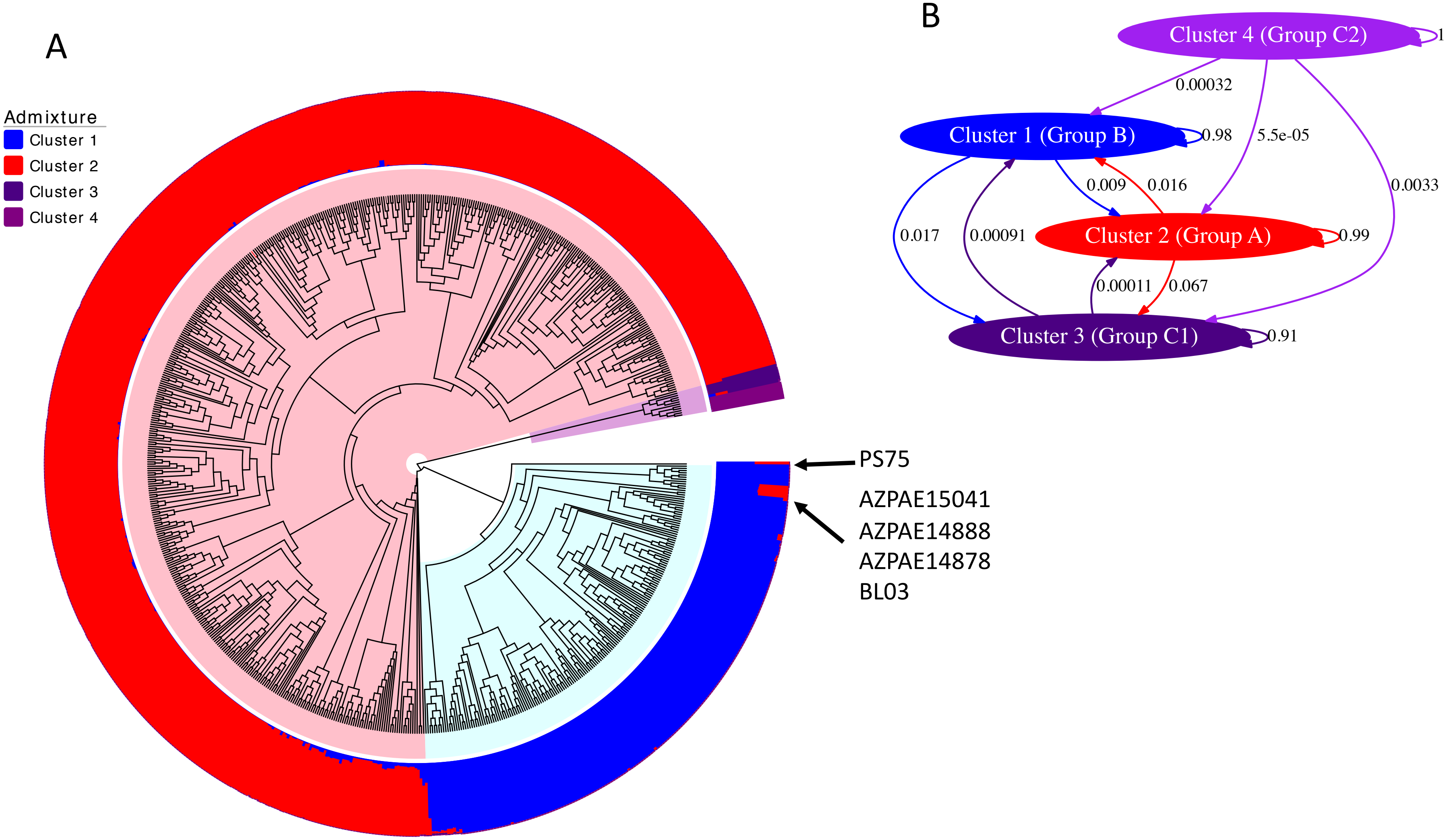

Supplemental Figure 5

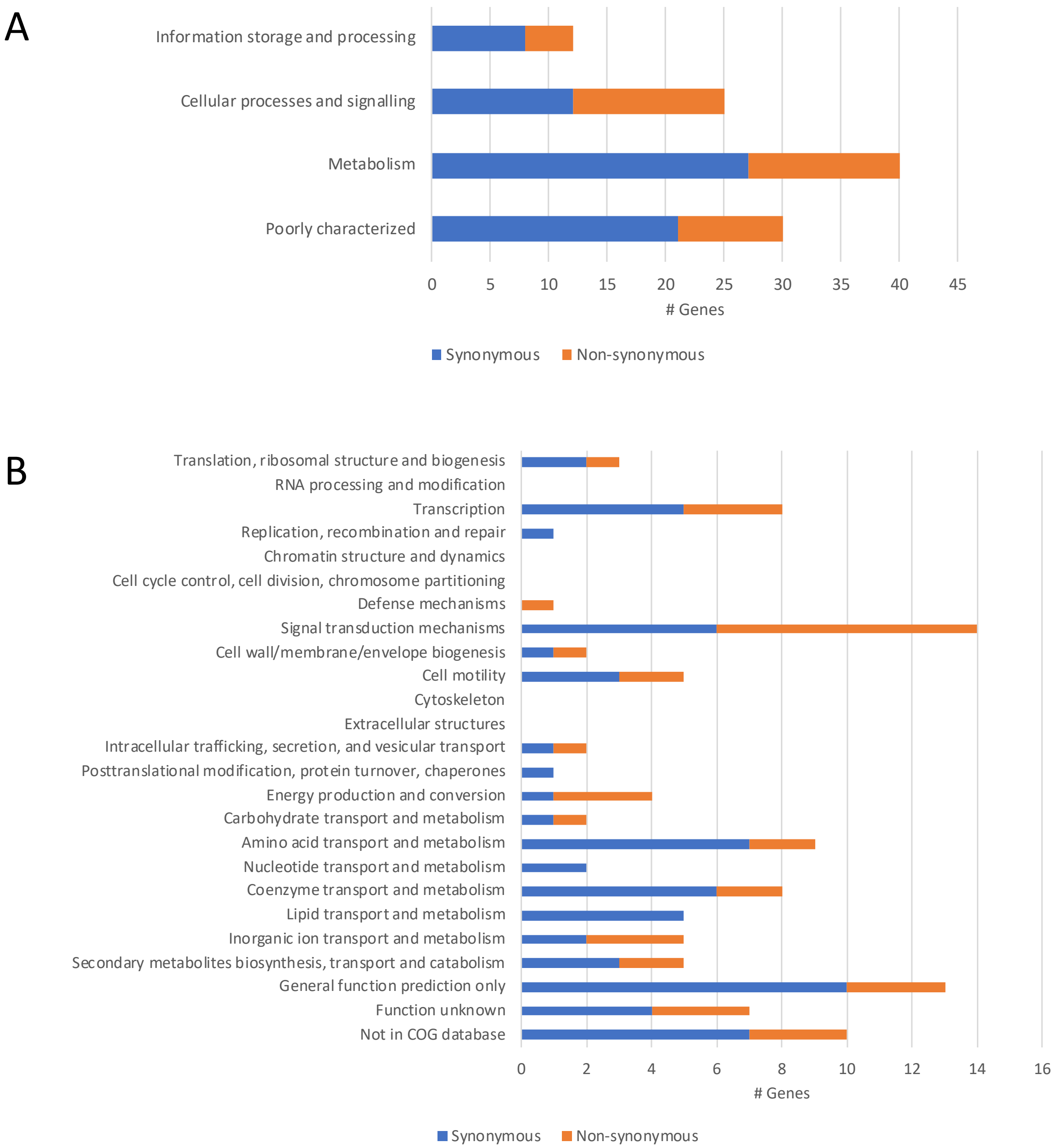

Supplemental Figure 6

A

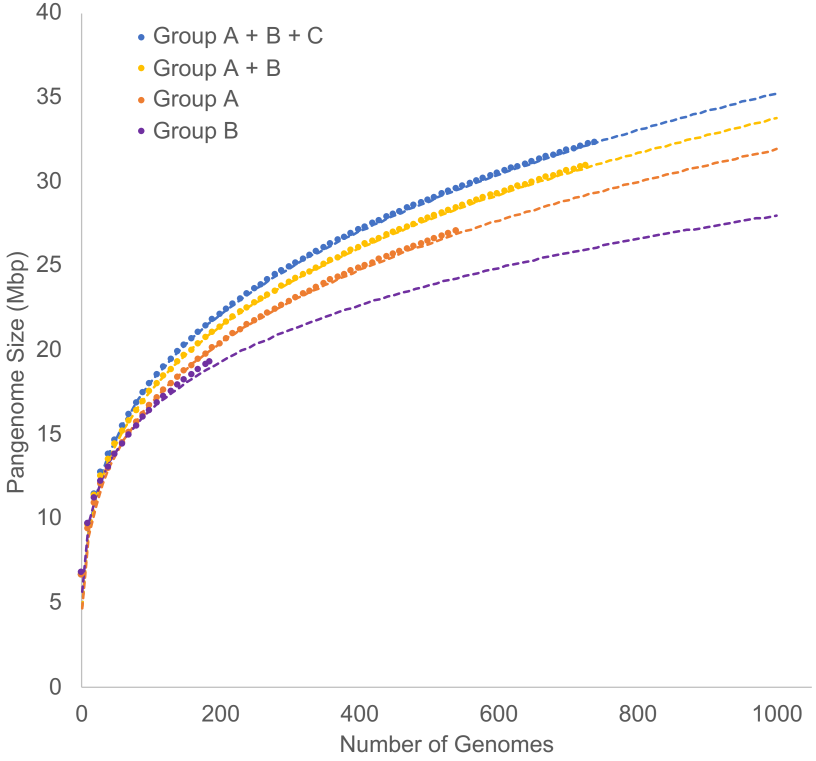

B

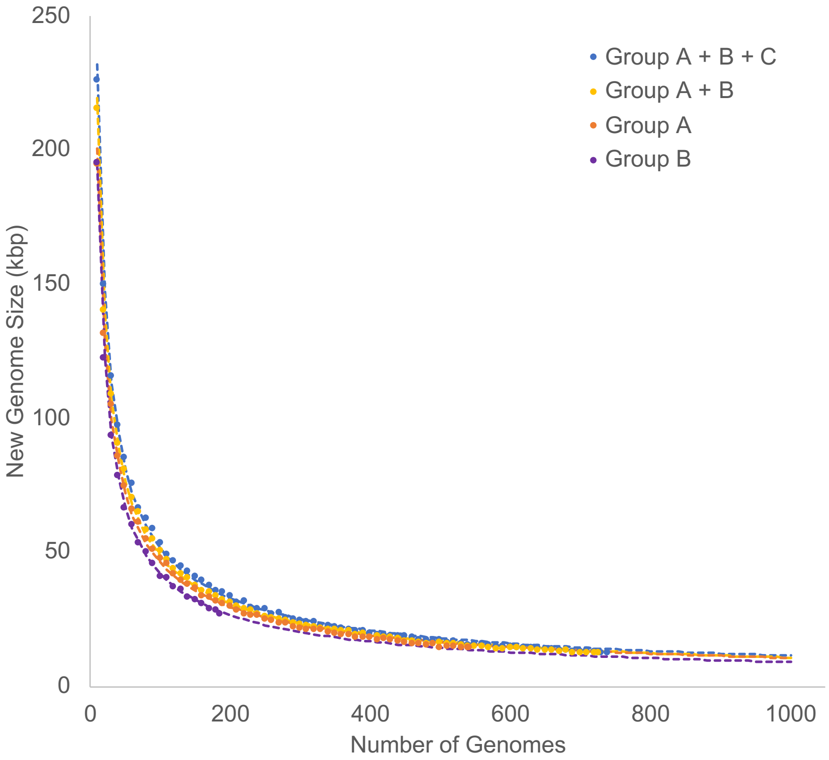

C

	a	b	Correlation R^2
Group A+B+C	0.6534	1045000	0.9978
Group A+B	0.6559	994700	0.9985
Group A	0.6383	872000	0.9972
Group B	0.6660	903100	0.9997

Supplemental Figure 7

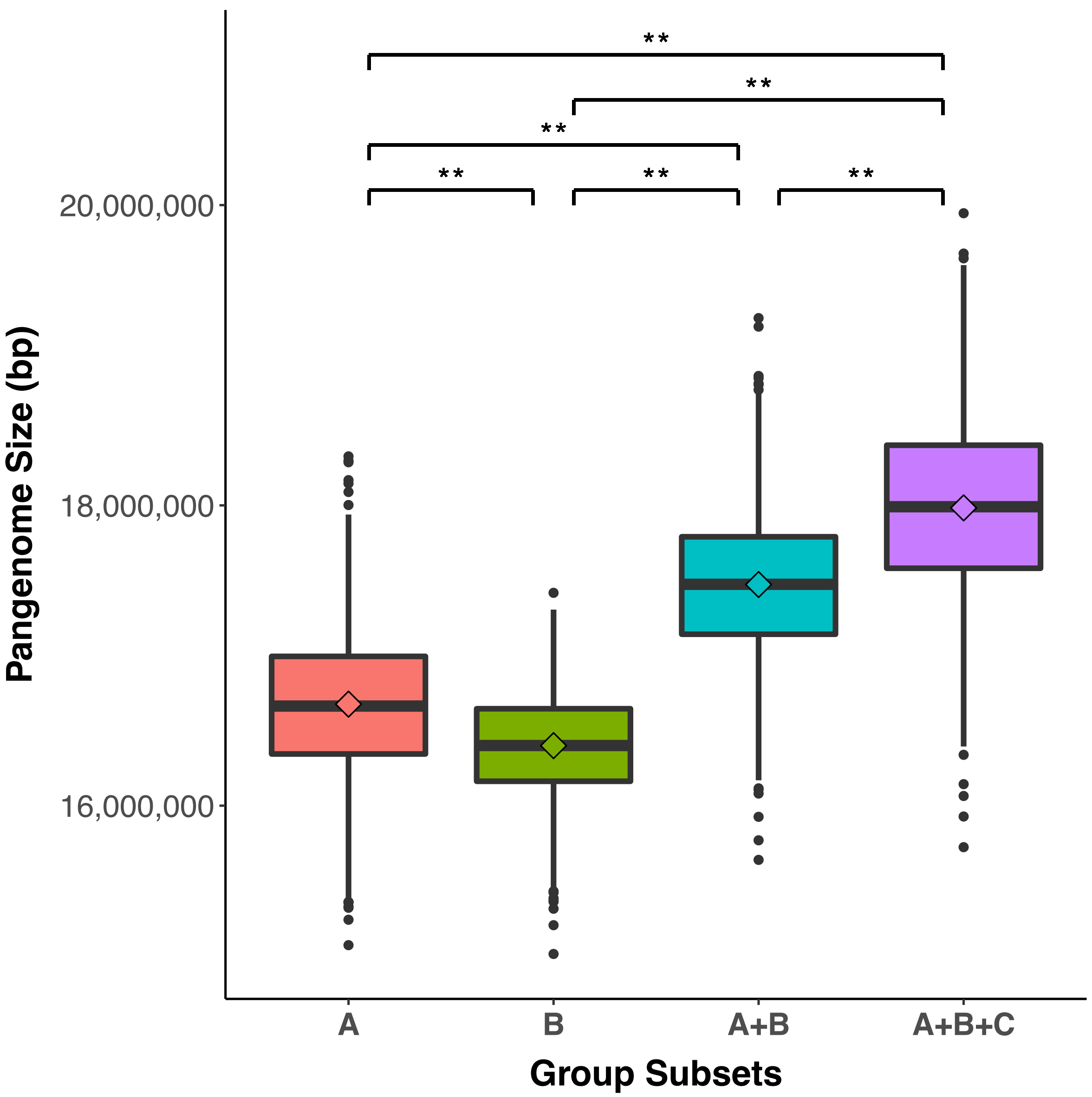

Supplement: Supplementary_Material_evz119 [file supplementary_material_evz119.zip › PApopulation_GenomeBio_20190521_Supplemental_Legend_Figures.pdf]
